# Efficacy and safety of acupuncture at a single BL1 acupoint in the treatment of moderate to severe dry eye disease: Protocol for a randomized, controlled trial

**DOI:** 10.1097/MD.0000000000010924

**Published:** 2018-06-01

**Authors:** Xue Zhang, Zhishun Liu, Wentao Ding, Jun Zhang, Huan Shi, Wenzeng Zhu

**Affiliations:** a Department of Acupuncture, South Area of Guang’anmen Hospital; b Department of Acupuncture, Guang’anmen Hospital; c Department of Ophthalmology, South Area of Guang’anmen Hospital, China Academy of Chinese Medical Sciences, Beijing, China.

**Keywords:** acupuncture, artificial tear, dry eye disease, RCT, study protocol

## Abstract

**Background:**

Dry eye disease (DED) is a very common disease. Although several current treatments offer some symptomatic relief, moderate to severe DED is still difficult to cure at present. Acupuncture may be effective for DED via its promotion of tear secretion and ability to improve symptoms, but evidence of its effectiveness is limited. The aim of this study is to evaluate the effect of acupuncture versus artificial tears in moderate to severe DED.

**Methods/design:**

A total of 120 participants with moderate to severe DED will be randomly assigned to either an acupuncture group or an artificial tear group at a ratio of 1:1. Participants in the acupuncture group will undergo treatments on a single BL1 acupuncture point 3 times per week for 8 weeks. Participants in the control group will use artificial tears 4 times per day for 8 weeks. The primary outcome will be the change from baseline in a mean Schirmer-I test (SIT) at week 8. The secondary outcomes will include the visual analog score (VAS) for the improvement of ocular symptoms, the ocular surface disease index (OSDI), the tear film break-up time (TBUT), and corneal fluorescein staining (CFS) at weeks 4, 8, and 32. Acupuncture acceptance will be evaluated at week 8. Adverse events will also be monitored and documented. A follow-up assessment will be conducted 24 weeks after the treatment.

**Discussion:**

We expect to verify that acupuncture is better than artificial tears toward improving the mean SIT values of patients with moderate to severe DED.

## Abstract 2

Supplemental Digital Content is available in the text

## 1 Introduction

Dry eye disease (DED) is defined as a tear and ocular surface disease caused by the poor quality and quantity of tear fluid, and the symptoms include ocular surface discomfort, visual acuity and tear film instability, and potential ocular surface damage, classified as aqueous-deficient dry eye and evaporative dry eye.^[1]^ The estimated prevalence of DED ranges from 5% to 35% in various regions, and the incidence of DED in China reached up to about 21% to 30%.^[2,3]^ In recent years, the number of DED patients has consistently increased, and it has become more common as more people spend time on staring at computer or mobile phone screens.^[4]^ DED not only causes a significant economic burden (including drug, follow-up, and recheck costs) but also leads to inconveniences in life, a reduced work efficiency and a reduced quality of life.^[5,6]^ Moreover, moderate-to-severe DED is considered to be similar to dialysis, severe angina, and hip fractures and is even more burdensome than these diseases.^[7]^ Therefore, DED has become an increasing concern worldwide in recent years.

The current medical strategies that address the DED-related symptoms mainly rely on artificial tears and lubricating gels, but this approach provides only short-term symptomatic relief.^[8–10]^ Because the underlying pathogenesis of DED has not been sufficiently addressed, there is no effective treatment for severe DED.^[11,12]^ In addition, the relief from DED-related symptoms that is achieved by artificial tears (AT) or lubricating gels is mainly temporarily palliative because these methods cannot improve tear gland function in these patients.^[13]^ With the further understanding of dry eye pathophysiology, the optimal intervention has shifted from simply lubricating the ocular surface by AT to promote the secretion of tears in a variety of ways.^[14]^ Thus, based on recent information, more conservative methods, especially that can increase tear secretion are needed for the treatment of DED.

Acupuncture has been developed to improve tear secretion, eye discomfort, and visual function in moderate-to-severe ocular inflammatory diseases, including DED. Several studies have reported the benefits of acupuncture for DED.^[15–17]^ The results of 2 systematic reviews of randomized controlled trials (RCT) have provided limited evidence on the effectiveness of acupuncture for treating DED;^[18,19]^ nevertheless, the strength of the evidence is suboptimal. Thus, no clear clinical evidence exists to support the application of acupuncture in the treatment of DED according to the guideline for the diagnosis and treatment of DED from the International Dry Eye WorkShop (DEWS). Thus, higher quality studies are needed to clarify the effects of acupuncture in DED patients.

This study is an RCT that evaluates the effectiveness of using acupuncture at a single BL1 acupuncture point versus artificial tears for the treatment of moderate to severe DED. We explore the ability of acupuncture to promote tear secretion, improve multiple eye discomfort symptoms and reduce ocular surface damage at the end of the treatment (8 weeks) and follow-up period (24 weeks after treatment). We also assess the acceptance of acupuncture compared with that of artificial tears and examine reports of adverse events (AEs).

## 2 Methods

### 2.1 Study aims

In this study, the efficacy of acupuncture for improving tear secretion and other symptoms of patients with moderate to severe DED versus artificial tears will be evaluated.

### 2.2 Study design

**Figure 1:**
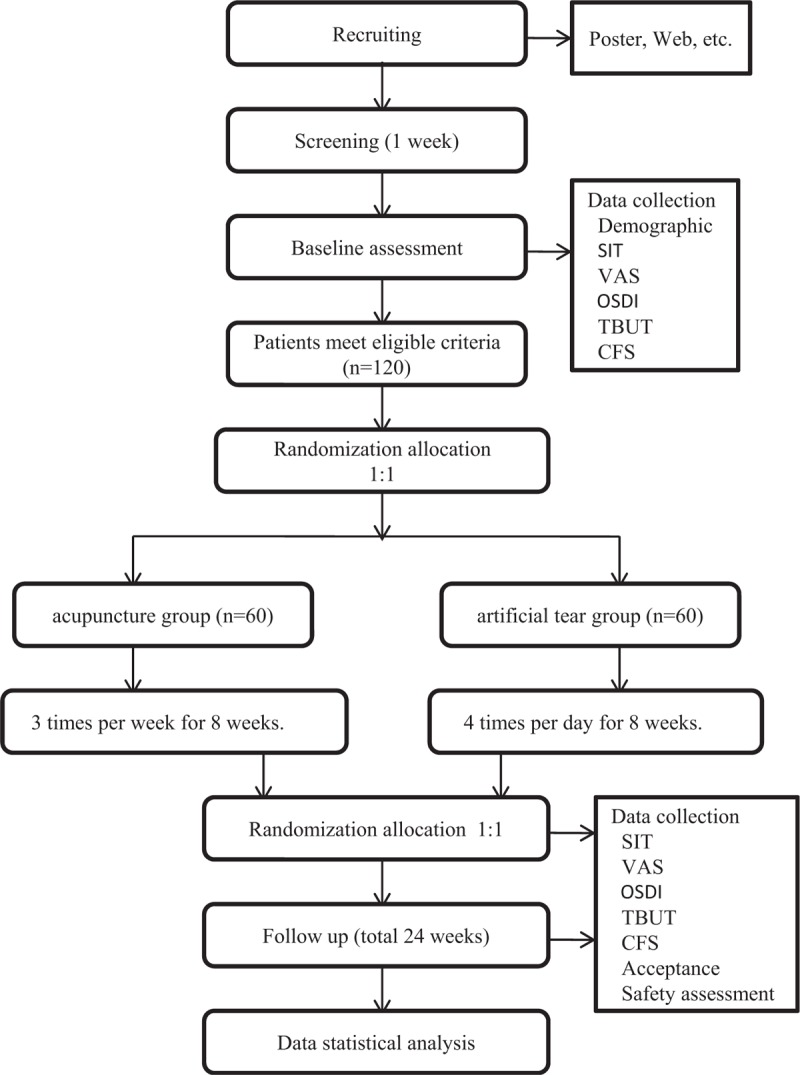
Diagram of trial design.

This is a single-center, prospective, controlled, randomized trial conducted in patients with moderate to severe DED. The flow chart is shown in Figure 1. We have developed the design and protocol based on the guidance for the Standard Protocol Items: Recommendations for Interventional Trials (SPIRIT) and the Standards for Reporting Interventions in Clinical Trials of Acupuncture (STRICTA).^[20,21]^

### 2.3 Study setting and recruitment

**Figure 2:**
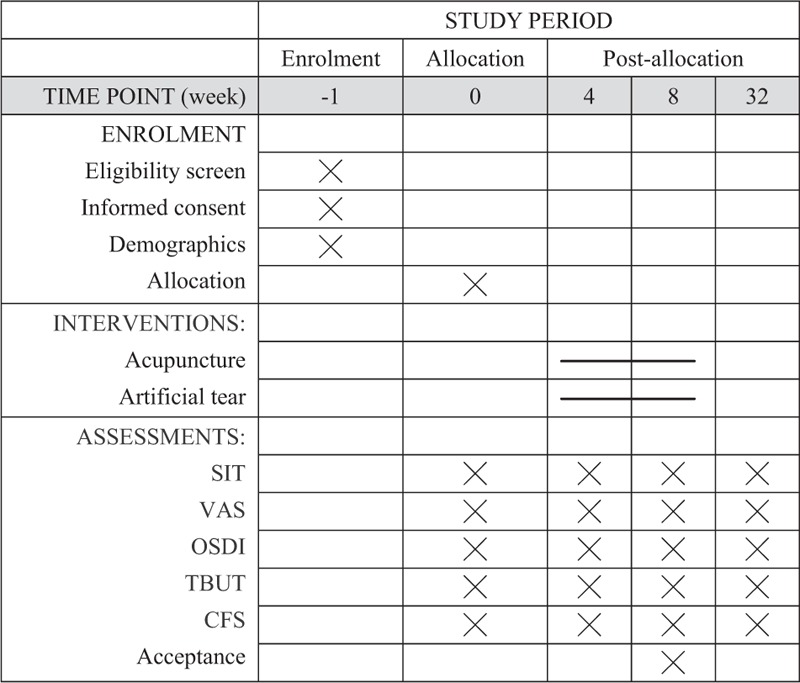
Standard protocol items: recommendation for interventional trials (SPIRIT) schedule of enrolment, interventions, and assessments. SPIRIT = standard protocol items: recommendation for interventional trials

The study will commence on September 1, 2018, and will be completed by December 31, 2019, at the South Area of Guang’anmen Hospital, China Academy of Chinese Medical Sciences. DED is diagnosed according to the criteria of International DEWS by an experienced ophthalmologist.^[22]^ The participants identified as eligible will be randomized into 2 groups—acupuncture and medicine—at a ratio of 1:1. The flowchart of the trial is shown in Figure 1. The total study period for this trial is 33 weeks (Fig. 2), including a 1-week baseline assessment, 8-week treatment period and 24-week follow-up period.

### 2.4 Inclusion criteria

The inclusion criteria will be as follows.

(1) Individuals will be > 18 years of age and < 60 years of age.
(2) Participants will meet the diagnostic criteria of DED.^[22]^ The criteria of moderate-to-severe DED are based on the following ophthalmological tests: a Schirmer-I test (SIT) (with the application of cocaine, a local anesthetic) value <10 mm/5 minutes, a tear film break-up time (TBUT) <10 seconds, and corneal fluorescein staining (CFS) ≥ 1.^[2]^ These ophthalmological tests will be performed by the ophthalmologists who do not know the results of the random allocation.
(3) Only the single eye with more severe scores or symptoms will be selected to observe the outcome.
(4) Participants who can complete the study, treatment, and assessments will be selected.

### 2.5 Exclusion criteria

The exclusion criteria will be as follows:

(1) participants with other ocular diseases (e.g., lacrimal duct obstruction, other conjunctiva and keratopathy) or immune system diseases (e.g., Sjogren syndrome and rheumatoid arthritis)
(2) participants with a history of surgery for DED within 3 months
(3) participants with cardiovascular, kidney, liver, or hematopoietic system diseases, mental health disorders, cancer or other severe diseases for whom acupuncture may be inappropriate or unsafe
(4) participants who received acupuncture within the past week
(5) women who are pregnant or lactating
(6) individuals who are participating in other clinical trials; and
(7) participants with a pacemaker, metal allergy, or severe fear of needles.

### 2.6 Randomization and blinding

The randomization will be performed by the Drug Clinical Trial Office affiliated with Guang’anmen Hospital using a computerized random number generator. Opaque, sealed envelopes will be numbered consecutively, and all sealed envelopes will be maintained by a researcher who is not involved in the treatment procedure or data analysis. After informed consent is obtained, an envelope will be opened by the researcher according to the participant's order of entry into the trial, and the assigned treatment will be offered to the participant. Two copies of the envelopes will be maintained to prevent the researchers from deviating from the randomization.

### 2.7 Intervention

#### 2.7.1 Acupuncture group

**Figure 3:**
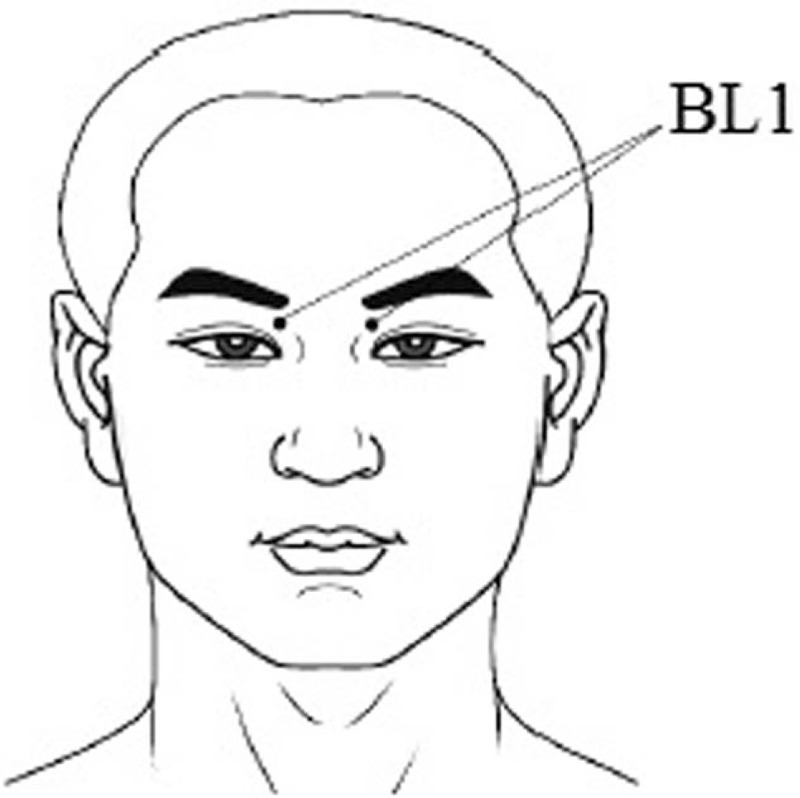
Location of acupuncture points.

The treatments will be initiated 1 week after participant randomization. Huatuo Brand stainless-steel needles (0.3 × 40 mm, Suzhou Medical Appliance Factory in China, CL) will be used in the acupuncture group. The acupuncture procedures are performed in accordance with the STRICTA guidelines.^[23]^ Acupuncture will be performed by a trained clinician with > 5 years of experience with acupuncture manipulation. The acupuncture regimen is based on our own clinical experience and pilot trial. The acupuncture point of the bilateral jingming (BL1), which is located on the face, in the depression between the superomedial parts of the inner canthus of the eye and the medial wall of the orbit, will be included in the acupuncture group (Fig. 3). After the participant sits on the chair, the acupuncturist will raise the participant's upper eyelid to expose the medial canthus mound (lacrimal caruncle). The needle will be vertically inserted rapidly into the BL1 point from the medial eyelid to a depth of approximately 0.5 inches. Then, the acupuncturist will vertically lift the needle for 1 minute, and the frequency will be approximately once every 3 seconds. The “deqi” sensation will be induced by lifting acupuncture. At this time, a large amount of tear fluid is expected to flow out of the participant's eyes. The acupuncturist will draw the needle out from the acupuncture point at this time. The treatments will be performed 3 times (Monday, Wednesday, and Friday) per week for 8 weeks and an average interval of 2 days between the 2 adjacent treatments.

#### 2.7.2 Artificial tear group

The control group will use sodium hyaluronate eye drops (Sodium Hyaluronate Eye Drops, EUSAN GmbH, Germany). The participants will be treated with 1 drop 4 times per day in the early morning, at noon, in the afternoon, and before bedtime for 8 weeks consecutively, with an average interval of 6 hours. A recording diary of the frequency and quantity of eye drops used in the trial will be collected at each visit.

In the 2 groups, other treatments, including different types of artificial tears, drugs, and alternative treatment (such as herbal medicine, acupuncture and cupping treatment) for DED will be banned during the treatment period. However, the participants will be allowed to use any treatment method of DED during the follow-up period. The usage of the treatments will be recorded by the participants and will be reported to researchers at the follow-up visit.

### 2.8 Outcome measures

#### 2.8.1 Primary outcome measure

The primary efficacy endpoint will be the increase from baseline in the SIT value with anesthesia at week 8. The SIT is a diagnostic method used to measure tear secretion,^[24]^ which is measured using tear secretion test paper (Color Bar Schirmer tear test, Eagle Vision, Inc., TN). First, the ophthalmologist responsible for the SIT will use local anesthesia (Alcaine Alcon, Inc., Texas) to arrest reflex tearing. Then, the participants will be requested to look above the eyes, and the ophthalmologists will place the tear secretion test paper under the lower eyelid. Last, the participants will keep their eyes closed for 5 minutes, after which the length of the wet test paper will be measured. The value of SIT equals the length of the wet portion.

#### 2.8.2 Secondary outcome measures

The following secondary outcome measures will be determined.

(1) The SIT with anesthesia will be measured at baseline, week 4, and week 32. Tear secretion is an important indicator that reflects tear secretion, and thus, we will also measure the value of SIT with anesthesia during the 32-week follow-up time. The SIT value can reflect the long-term effect of acupuncture on the improvement of tear secretion.
(2) The symptoms of DED will be assessed by the participant using a self-administered VAS ranging from 0 to 100 based on recognized DED symptoms (eye dryness, itching, sticky feeling, photophobia, pain, stinging or burning, sandy feeling or grittiness, or a foreign body sensation) at baseline, week 4, week 8, and week 32. The means of these 10 VAS scores will be calculated and used as a global symptoms score.
(3) The OSDI will be used to assess the influence of DED on the participant's life at baseline, week 4, week 8, and week 32. The OSDI is a valid and reliable questionnaire and has been used widely in clinical trials.^[25]^ The questionnaire includes 12 problems that reflect vision-related function and environmental stimuli. The total scores will be between 0 and 100. Higher scores represent a more severe dry eye state.
(4) The TBUT will be measured at baseline, week 4, week 8, and week 32. The TBUT test is used to assess tear film stability.^[26,27]^ After staining with sodium fluorescein, the first dry spot of the tear film will be observed with a slit lamp and the time of appearance will be measured. The time will be measured repeatedly 3 times, and the average time of the 3 measurements will be the TBUT. A TBUT < 10 seconds will suggest that moderate to severe DED.
(5) CFS, which is used to evaluate ocular surface damage and monitor the clinical response to therapy, will be measured at baseline, week 4, week 8, and week 32. Corneal staining will be examined under standard illumination with a slit lamp using a cobalt blue filter and will be graded using the modified Oxford scale.^[28]^
(6) To investigate which treatment is preferred, acupuncture or artificial tear acceptance will be assessed at week 8. A 4-point scale will be used, with 1 representing “very difficult to accept,” 2 representing “difficult to accept,” 3 representing “easy to accept,” and 4 representing “very easy to accept.”^[29]^
(7) AEs will be assessed using a questionnaire at the end of the treatment and follow-up periods. All participants will be requested to report AEs during the treatment and follow-up periods. All AEs will be assessed for severity (mild, moderate, severe) and seriousness by the investigator.

### 2.9 Data collection

The data collection will be from the case report forms recorded by the investigator in the trial. The demographic, clinical, and examination results of DED from the participants will be recorded at baseline and during the treatment and follow-up periods. Moderate-to-severe DED will be diagnosed by an experienced ophthalmologist after an ophthalmology examination. The investigator will enter all of the collected data into the case report forms. At baseline, during the treatment period (8th week) and during the follow-up period (32nd week), the forms concerning the questionnaire survey will be completed by the participants under the guidance of a full-time staff member; other forms concerning the ophthalmology examination will be filled in by the investigator according to objective examination results.

### 2.10 Sample size and statistical analysis

The sample size calculation is based on the mean SIT value. According to our pilot trial,^[30]^ the decrease in the mean SIT value in the acupuncture group at week 8 is 3.41 ± 3.04. Based on a previous report, the mean value of the decreases in the artificial tear group is 1.8 ± 1.6.^[2]^ We have used PASS Version 11.0 (International Business Machines Corporation, China) software to calculate a sample size of 120 for each group to provide 90% power to detect a difference of 1.61 between the groups with a 2-sided 5% level of significance, allowing for a 20% dropout rate, and with the participants receiving the treatments and completing the follow-up (60 participants in each group).

SPSS Version 22.0 (International Business Machines Corporation, China) software will be used to perform the statistical analysis. Two-sided tests will be used for all statistical analyses. The level of significance is established at 0.05. Efficiency and safety analyses will be based on all participants at randomization according to the intention-to-treat (ITT) principle. All participants who have accepted randomization will be included in the statistical analysis of data. All original data collected from participants in the trial will be included in the statistical analysis. The data cannot be changed or eliminated at random. The missing data in the case report forms will be replaced randomly by the multiple imputation values.^[31]^

We will use the mean and standard deviation (SD) to describe continuous data if the data have a normal distribution and the mean and 95% confidence interval (CI) to describe continuous data if the data are skewed. We will use the percentage or 95% CI to describe categorical data. For comparisons with baseline data, we will use a paired *t*-test for continuous data and a nonparametric test for categorical data. For comparisons of the differences between the 2groups, T-tests or Mann–Whitney *U* tests will be used for comparisons between continuous variables, and chi-square tests or Fisher's exact tests will be used for comparisons between categorical variables, as appropriate. For assessing safety in the trial, the investigator will present the AEs and adverse reactions with tables, which will comprise the categories of AEs, the severity of AEs, the incidence of AEs, and correlation with the treatment. Every participant with serious AEs will stop receiving the intervention. All details will be reported in a timely manner.

### 2.11 Quality control

The acupuncture practitioners responsible for the acupuncture treatments must have a minimum of 5 years of clinical experience. All of the investigators will take a one-day training course for this trial. This training course will cover the study protocol, acupuncture operation, precautions for drug use, the recording method for CRF, disposal of bleeding, a researcher duties and research monitoring. Full-time researchers who are not aware of the random allocation of groups will be responsible for the assessments of all scales. During the trial, supervisors will check on case reports and acupuncture treatments once a month. Drop-outs, withdrawals (with the reasons), and the compliance of all participants will be recorded in detail throughout the treatment and follow-up period. Two postgraduates (not involved in the trial) will input the data independently and verify the database together.

### 2.12 Ethics and dissemination

The study protocol is in accordance with the principles of the Declaration of Helsinki,^[32]^ was approved by the Ethics Committee of Guang’anmen Hospital of China Academy of Chinese Medical Sciences (approval number 2018-012-KY-01) on April 19, 2018 and was registered on April 23, 2018, at http://www.chictr.org.cn/ (ref. ChiCTR1800015831). All important protocol modifications will be submitted to the Ethics Committee of Guang’anmen Hospital. The researchers will inform all participants of the potential risks and benefits of the study. Informed consent will be obtained for all participants from either the participant or the participant's legal representative. All participants will be required to be able to understand written instructions, complete the examination and assessment forms. The personal information of all participants will be hidden from other users and stored in a specific cabinet with a lock. Statistics and monitoring managers will have the right to obtain the final study data set. To prevent the participants from suffering harm from trial participation, we will provide corresponding treatment and medical consultations with the ophthalmologist for free. We will disseminate the results of this study in meetings or publications when the trial is completed.

### 2.13 Study organization and funding

This trial is supported by the South Area of Guang’anmen Hospital, China Academy of Chinese Medical Sciences fund (Funding no. Y2017-11).

## 3 Discussion

The aim of this study is to evaluate the efficacy and safety of using acupuncture at a single BL1 acupuncture point for improving tear secretion and other symptoms of patients with moderate to severe DED. Two systematic reviews and several previous studies have shown that acupuncture is effective in the treatment of DED. For example, Jeon et al conducted an observational study on the acupuncture treatment of DED in the Korean Oriental medicine hospital.^[16]^ The results of the trial showed that acupuncture treatment could effectively improve the symptoms of DED and tear secretion. In a clinical study comparing the effects of acupuncture and artificial tears on DED, which was conducted by Kim et al^[15]^ in South Korea, a better curative effect was observed during the 8-week follow-up period in the acupuncture group. Thus, to reveal whether acupuncture treatment has a longer lasting effect than artificial tear treatment, our trial will carry out a 24-week follow-up. Although several studies have shown that acupuncture may be effective for DED, a negative result of a clinical study has been reported. A randomized, double-blind trial with acupuncture versus sham acupuncture for DED was conducted by Shin et al^[33]^ in the Korea Oriental medical hospital. After 4 weeks of treatment, the trial results showed that acupuncture did not offer any significant improvement over sham acupuncture. The result of the trial suggests that the effect of acupuncture in the treatment of DED is controversial. Different results may occur due to different acupuncture points, acupuncture manipulation, primary outcomes, treatment duration, outcome measures, severity of disease, sample sizes, or other factors.

The main difference from previous studies is that only a single acupuncture point will be used. In this study, we will not use the conventional acupuncture manipulation, but instead of puncturing the BL1 acupuncture point from the inside of the eyelid to a depth of 0.5 inches. A large amount of tear fluid flows out of the eyes of the participants after each acupuncture treatment, which can guarantee a curative effect. The second difference from previous studies is that the primary outcome of our study is the measurement of tear secretion with the SIT. The most obvious advantage of our acupuncture at a single acupuncture point by special acupuncture manipulation in this study is to promote the tear secretion. Third, this study will involve a treatment duration of 8 weeks, which is an appropriate treatment duration for acupuncture therapy for moderate to severe DED. Moreover, according to the suggestion of the DEWS, any clinical study that involves the treatment of DED with artificial tears should be performed for at least 6 to 8 weeks. Fourth, acupuncture manipulation of that point will only last for 1 minute in our trial, which is different from other acupuncture operation for 20 to 30 minutes. The use of this acupuncture method in the treatment of DED may be more effective and convenient based on our previous clinical experience.

The characteristics of DED include reduced tear secretion and ocular surface damage. It is generally believed that the obstruction of tear secretion is an important pathogenesis of DED.^[34]^ The mechanism of acupuncture at the BL1 point for the treatment of DED is derived from the anatomy and physiology of acupuncture points. According to the anatomical structure, there are lacrimal sacs and lacrimal canaliculi below BL1, which can stimulate tear secretion via acupuncture and manipulation. To achieve a certain depth of acupuncture, the needles will be inserted into BL1 from the inside of the eyelid. Moreover, repeated acupuncture manipulation can improve the function of the lacrimal sac to secrete tears, which explains the persistent long-term effect after acupuncture treatment.

There are several limitations in our trial. First, the trial is conducted in a single center. Thus, regional differences cannot be shown. Second, because the trial will be conducted in the hospital of traditional Chinese medicine, many participants may have a higher expectation of acupuncture therapy. Third, participant blinding is impossible due to wide differences between acupuncture and artificial tear treatments. Fourth, we are not conducting a placebo control trial because we consider that the use of sham acupuncture may not provide sufficient sensitivity and may not meet ethical guidelines.

## Acknowledgments

All authors are sincerely grateful for the editorial work of American Journal Experts.

## Author contributions

Authorship: ZX, ZWZ and LZS conceived of the idea and design of the study. ZX drafted and edited the final manuscript for submission. DWT and SH contributed to the data collection. ZWZ contributed to supervision and reviewed the final paper. SH and DWT prepared the related information sheets, consent forms, and case report forms. ZJ contributed to resources and validation. All authors approved the final manuscript.

**Conceptualization:** xue zhang, Zhishun Liu, Wenzeng Zhu.

**Data curation:** WenTao Ding, Huan Shi.

**Formal analysis:** xue zhang.

**Funding acquisition:** xue zhang.

**Investigation:** xue zhang, WenTao Ding, Huan Shi.

**Methodology:** Zhishun Liu, Wenzeng Zhu.

**Project administration:** Wenzeng Zhu.

**Resources:** Jun Zhang.

**Supervision:** Wenzeng Zhu, Jun Zhang.

**Validation:** Jun Zhang.

**Writing – original draft:** xue zhang.

**Writing – review & editing:** Zhishun Liu, Wenzeng Zhu.

## Supplementary Material

Supplemental Digital Content
